# A Narrative Literature Review of Natural Language Processing Applied to the Occupational Exposome

**DOI:** 10.3390/ijerph19148544

**Published:** 2022-07-13

**Authors:** Annika M. Schoene, Ioannis Basinas, Martie van Tongeren, Sophia Ananiadou

**Affiliations:** 1 Department of Computer Science, University of Manchester, Manchester M13 9PL, UK; 2 Department of Health Science, University of Manchester, Manchester M13 9PL, UK; ioannis.basinas@manchester.ac.uk (I.B.); martie.j.van-tongeren@manchester.ac.uk (M.v.T.)

**Keywords:** natural language processing, exposure research, exposome, machine learning

## Abstract

The evolution of the Exposome concept revolutionised the research in exposure assessment and epidemiology by introducing the need for a more holistic approach on the exploration of the relationship between the environment and disease. At the same time, further and more dramatic changes have also occurred on the working environment, adding to the already existing dynamic nature of it. Natural Language Processing (NLP) refers to a collection of methods for identifying, reading, extracting and untimely transforming large collections of language. In this work, we aim to give an overview of how NLP has successfully been applied thus far in Exposome research. Methods: We conduct a literature search on PubMed, Scopus and Web of Science for scientific articles published between 2011 and 2021. We use both quantitative and qualitative methods to screen papers and provide insights into the inclusion and exclusion criteria. We outline our approach for article selection and provide an overview of our findings. This is followed by a more detailed insight into selected articles. Results: Overall, 6420 articles were screened for the suitability of this review, where we review 37 articles in depth. Finally, we discuss future avenues of research and outline challenges in existing work. Conclusions: Our results show that (i) there has been an increase in articles published that focus on applying NLP to exposure and epidemiology research, (ii) most work uses existing NLP tools and (iii) traditional machine learning is the most popular approach.

## 1. Introduction

Natural Language Processing is an area of research within Artificial Intelligence (AI) that is concerned with giving computers the ability to understand natural language (spoken and written) in the same way a human could [1]. Knowledge of computational linguistics (rule-based modelling of human language), statistics, machine learning and deep learning are used either individually or combined to achieve the aforementioned goal [2]. The term *Exposome* was first introduced by [3], who defined an area of research that takes systematic measurements of exposures (e.g., occupational, physical environment or socio-economic factors) that a person is exposed to throughout life (pre-birth until death) and affects their health outcomes [3]. However, the term *Exposome* itself has not been fully integrated into all areas of exposure research yet, where often the term ‘*exposure research*’ is used when referring to the same or similar concepts [4]. At the same time, text mining and NLP techniques are increasingly applied in a variety of exposure-related research areas. Whilst there are a variety of surveys and literature reviews in NLP and its various subtasks [5,6,7], there is no review of NLP and text mining techniques used in the field of occupational and environmental exposure research. This review fills that gap by providing a description of existing tools based on NLP and text mining techniques that have been applied in occupational and environmental exposure research. For this, we utilise a hybrid approach combining classical and automatic reviewing methods with RobotAnalyst [8], which is a recently developed web-based software system that combines text mining and machine learning algorithms. Papers published in the PubMed, Scopus and WoS databases are screened and reviewed to answer the following research questions:

- What are the most common text mining and NLP approaches used in exposure assessment research?
- What resources are used for this task?
- What are the most common NLP methods used?
- What are the main challenges and future directions of research?

## 2. Review Methodology

**Figure 1:**
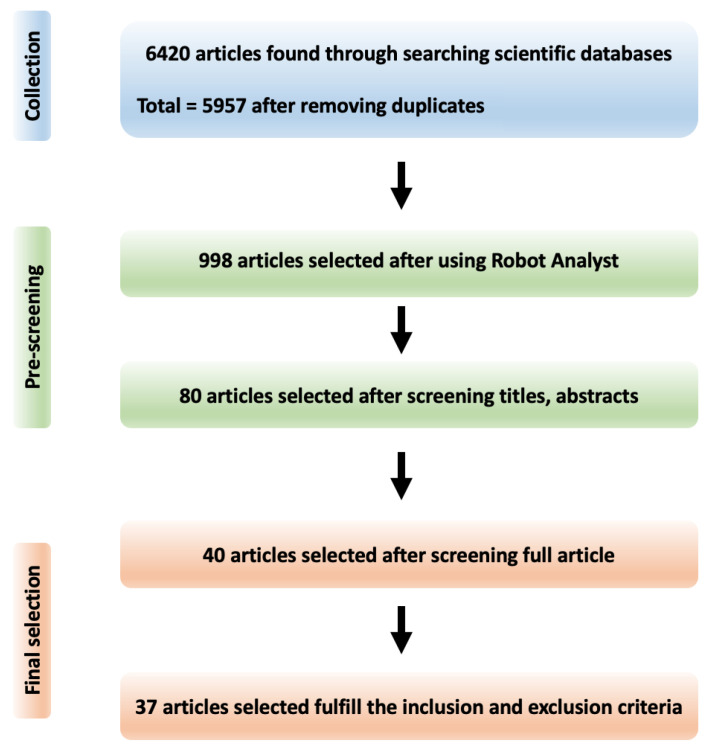
Overview of article selection process used in this narrative literature review.

In this literature review, a search was conducted in three scientific literature databases. We include articles available in full and peer-reviewed, where our search returned 6420 articles, out of which 5957 were selected for pre-screening after duplicates were removed. In Figure 1, we show the process of selecting for this review, where for each search on the three different platforms (PubMed, Scopus and Web of Sciene), we used the following query terms:

(“natural language processing” OR “text mining” OR “text-mining” OR “text and data mining” OR ontology OR lexic* OR corpus OR corpora) AND (exposome OR exposure OR socioexposome OR (“risk factor” AND (“work” OR “occupational” OR “environmental*”)))

Pre-screening was performed as a two-step process. First, to reduce human workload, we utilised RobotAnalyst [8] to identify 998 full papers. RobotAnalyst is a web-based and freely available software system that utilises both text mining and machine learning methods to categorise and rank references for their relevance (Free access to RobotAnalyst can be requested to reproduce this work: http://www.nactem.ac.uk/robotanalyst/ (accessed on 2 November 2021). The system uses an iterative classification process which makes decisions based on the abstract for each reference. Next, we manually screened the titles and abstracts of those papers using the inclusion and exclusion criteria outlined below. The inclusion and exclusion criteria used to select studies relevant to occupational exposure research were provided by two experts in occupational exposure. Based on these criteria, we identified 80 papers that specifically focused on text mining and/or natural language processing in the field of exposure research. Next, the full papers were reviewed for their relevance to occupational exposure and usage of NLP or text mining methods. Finally, 40 copies of the full papers of those were retrieved and reviewed in full, resulting in a total of 37 articles that fulfilled our defined inclusion and exclusion criteria.

**Inclusion criteria:**

- Original work;
- Study exposures concerning humans;
- Study occupational and/or environmental exposures of humans, such as airborne agents (e.g., particulates or substances and biological agents (viruses)), stressors, psycho-social and physical (e.g., muscle-skeletal) exposures as well as workplace accidents;
- Have their full texts available;
- Are written in English;
- Focus on text mining or natural language processing and their texts containing a method, experiments and result section.

**Exclusion criteria:**

- Studied animal or plant exposures;
- Studied drug, nutrition or dietary exposures on humans;
- Written in another language than English;
- Commentaries, opinion papers or editorials.

## 3. Results

**Figure 2:**
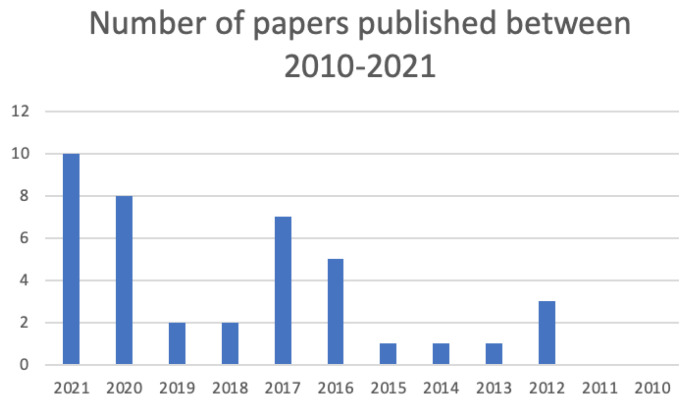
Number of NLP papers applied to occupational exposure research published each year from 2010 to 2021.

In the following section, we summarise the findings of this literature review, where we focus on the types of resources used, computational methods and existing NLP tools. In Figure 2, we show the number of papers published each year, where we can observe an increase in publications over time. We also categorise each paper in Table 1 based on NLP tools used, resources and computational method. Finally, we give a brief overview of the literature reviews and qualitative research in this area.

**A.**  **Resources**

**Figure 3:**
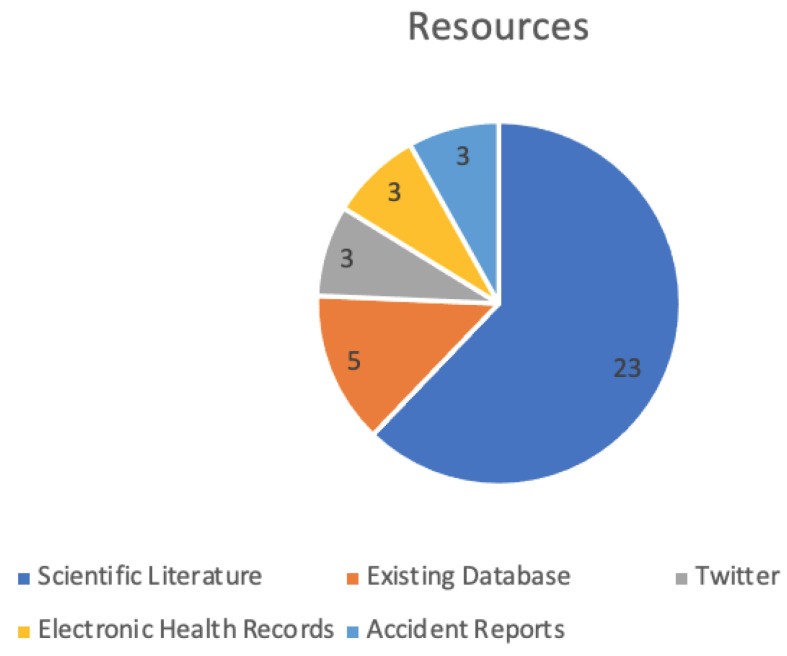
A chart showing the different types of resources used in the selected articles.

There are different types of resources used, where the most common resource is the existing scientific literature (see Figure 3). Other data sources include databases, social media platforms, electronic health records and accident reports (see Table 1).

**B.**  **Computational Methods**

**Figure 4:**
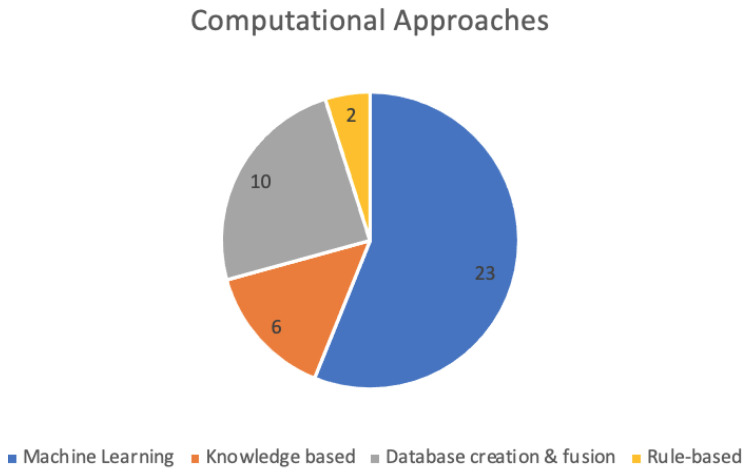
A chart showing the computational methods utilised in the selected articles.

Overall, there are four main categories of computational approaches used which include machine learning, knowledge-based approaches, and database creation and fusion approaches. Figure 4 shows the split of computational approaches found in this review.

**C.**  **Existing NLP tools**

**Figure 5:**
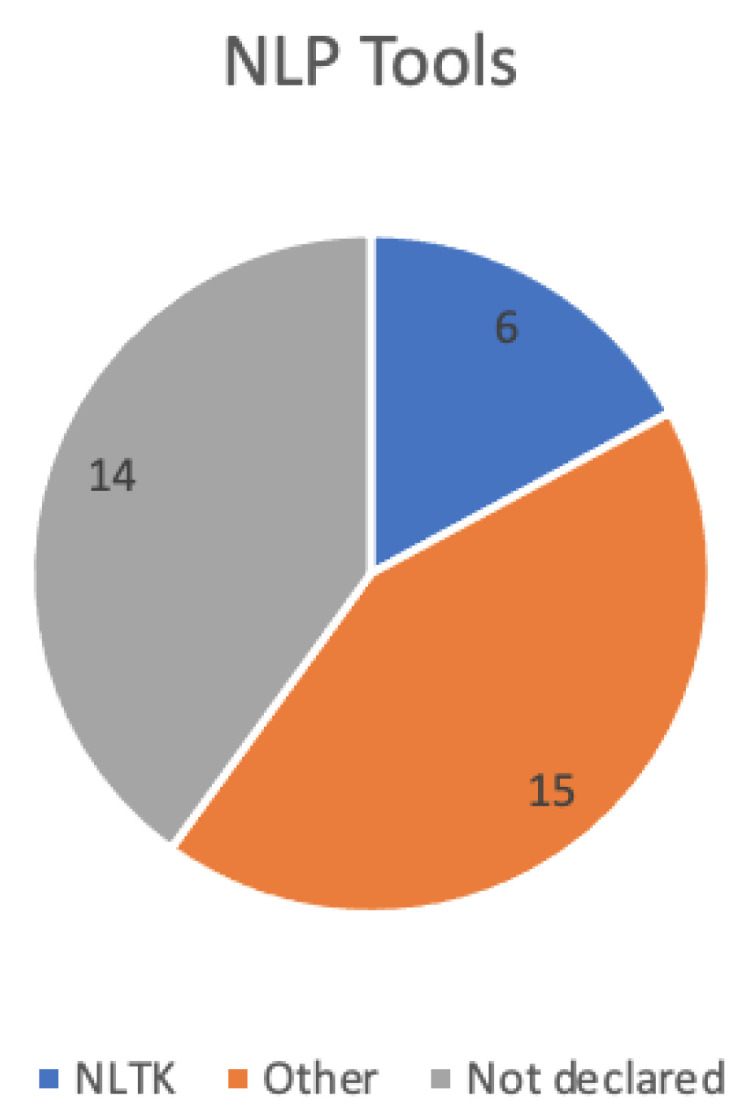
A chart showing a summary of the different types of NLP tools in each article.

There are a number of different existing NLP preprocessing tools used (see Figure 5), where NLTK [49] is the most commonly used for preprocessing textual data. Given the vast number of different NLP tools used in other studies, we have summarised the tools as ‘*Other*’. However, it has to be noted that a large amount of studies did not declare the type of text mining tool that was used in their work.

## 3.1. Machine Learning Methods

Ref. [9] proposes a contactless clinical decision support system to diagnose patients with COVID-19 and monitor quarantine progression using Electronic Health Records. Relevant keywords are extracted from unstructured text using NLTK, and the results are added to a searchable database. The final steps of this work include the integration of the system with cloud services and visualisation to make results accessible to clinicians. The work by [28] proposes a computational approach of mapping the impact of climate change on global health via scientific literature. A total of 3730 papers are labelled manually and subsequently fed into an SVM (Support Vector Machine) to classify the unlabelled documents into the different label categories. Next, topic modelling is used to analyse and visualise the content of the literature. The authors of [15] propose to use scientific literature on PubMed to assess the impact of environmental exposures from early life using different unsupervised learning methods (e.g., LDA (Latent Dirichlet Allocation)) to gain insight into the different topics. The work by [29] models the impact of COPD (chronic obstructive pulmonary) from smoking using Adverse Outcome Pathways generated from the scientific literature. The is collected and filtered from PubMed to create a corpus and then clustered using the text mining approach proposed by [50]. Research by [10] classifies incident reports to improve aviation safety into two categories using an LSTM (Long Short-Term Memory) with attention. A total of 200,000 reports are preprocessed using NLTK, and word vectors are generated using ULMFiT (Universal Language Model Fine-tuning for Text Classification) [51]. Ref. [12] extracts information from the scientific literature to evaluate the impact of human exposure to electromagnetic fields, where topic modelling is used to generate domain-specific lexicons. Work by [42] develops a computational literature review approach for in utero exposure to environmental pollutants, where they aim to identify multiple chemicals and their health effects and reduce the burden of manual literature reviews. The titles and abstracts of 54,134 papers are clustered using the DoCTER software [16]. The authors of [30] propose a network-based predictive model to assess chemical toxicity for risk assessment of environmental pollutants. The Registry of Toxic Effects of Chemical Substances (RTECS) database [52] is used, where chemicals were annotated with an identifier to show the structure of it. Work by [13] introduces a supervised machine learning approach to complement a previous manual literature retrieval for the Exposome-Explorer database [53], where an extensive variety of machine learning algorithms are evaluated using Sckit-Learn [54]. Ref. [48] uses multivariable logistic regression to classify the spread of household transmission of COVID-19 in healthcare workers. As part of this work, term-frequency inverse document frequency (tf-idf) matrices are used match confirmed cases by residential address. The authors of [17] use Chinese accident reports for safety risk analysis in the construction industry, where a software called ROST is used to preprocess the documents and perform cluster and network structural analysis. Research conducted by [14] develops a corpus of over 3500 abstracts that were manually annotated by an Exposome expert for chemical exposures according to a taxonomy. The taxonomy is based on 32 nodes and was split into two categories: biomonitoring and exposure routes. Finally, the data were fed into an SVM (Support Vector Machine) to classify unseen documents. The authors of [11] analyse the sentiment of tweets collected based on a specific geolocation (Texas counties along I-20) to determine if there is a link between CVD (cardiovascular disease) rates and factors that may cause or increase the risk included on the tweets. A voting classifier is used to determine the sentiment of each tweet into positive or negative, where an accuracy of 73.69% is achieved. Ref. [31] developed an ensemble classifier, called *SOCcer*, to map job titles to occupational classification codes (SOC). For this, a variety of publicly available resources were used to match job titles and tasks to the US SOC-2010 code, which resulted in a knowledge base of around 62,000 linked jobs. To train the ensemble classifier, job descriptions from a bladder cancer study were used as training data, whereas an evaluation of the algorithm was conducted on job titles for personal airborne measurements during an inspection. Research conducted by [18] collected data using Twitter’s API for ‘*asthma*’, and both manual (e.g., expert annotation and evaluation) and automatic analysis (e.g., topic modelling) are conducted to identify health-related tweets. One of the dominant topics identified by experts was environmental influences and references to triggers of asthma. The work by [22] uses text mining to assess chemical health risks, where PubMed abstracts are used to identify the mode of action (MOA) of carcinogens. For this work, they use the previously developed CRAB tool [55], which uses a bag-of-words approach to convert abstracts into vectors. Then, an SVM classifier with Jensen–Shannon divergence (JSD) kernel is trained to categorise the abstracts into a predefined taxonomy. The work by [23] develops a ranking algorithm to automatically recommend scientific abstracts for curation at CTD (Comparative Toxicogenomics Database [56]). This is completed by screening each abstract and assigning a document relevancy score (DRS), where 3583 articles are used from PubMed for this task. To analyse each abstract, a variety of text mining tools and approaches are used, which include ABNER [57], MetaMap [58] and Oscar3 [59] for gene/protein recognition and chemical recognition, respectively. Finally, a ranking algorithm is developed that sorts abstracts for curation relevance. The authors of [24] introduce a new method to classify biomedical documents for curation using the Comparative Toxicogenomics Database (CTD). A total of 1059 previously collected articles are annotated for entities (e.g., genes, chemicals, diseases and respective interactions), and manual abstract annotation is performed for chemicals relevant to the CTD. Finally, the documents are classified using a SVM. The authors of [25] use 225 electric power causality accident reports from China to identify factors that contribute to personal injury. TF-IDF is used to obtain the word frequency in a document, and the results are subsequently visualised using word clouds. The results are then used to extract key information on the dangers described in the reports. Our results also show that the majority of papers in this section utilise existing literature or databases to extract new information or classify unseen documents into existing categories. Classification experiments are performed using a wide variety of existing supervised machine learning algorithms (e.g.,: SVM or logistic regression). At the same time, new information is commonly uncovered and visualised using unsupervised learning methods (e.g.,: LSA or PCA). NLTK is a commonly used tool for preprocessing textual data, but there are also other NLP tools utilised that may be more suitable to deal with different languages or domains (e.g., ROST or CRAB).

## 3.2. Knowledge-Based Methods

Ref. [43] investigates Adverse Outcome Pathways (AOP) of pesticide exposure based on scientific literature collected on PubMed. For this, the recently developed AOP-helpFinder [60] is extended and subsequently known as AOP-helpFinder 2. The following properties were added: (i) the tool’s ability to automatically process and screen abstracts from PubMed, (ii) link stressors with a dictionary of events and (iii) calculate scores for both systems based on the position and weighted score for all event types. The tool is then evaluated by applying it to screen for a list of pesticides that have unknown long-term exposure effects on human health. Research conducted by [44] utilises a linguistic analysis of 261 scientific abstracts related to the ‘Exposome’ to gain insight into the current range of exposome research conducted. A literature search was performed, and an analysis was conducted using a combination of Termine [61] and NLTK [49] to extract multi-word terms and compute word frequency counts. The second part of this analysis uses over 500 biomedical ontologies provided at the National Center for Biomedical Ontology to automatically map abstracts to relevant ontologies. This work was subsequently extended by [62], who are using topic modelling and ontology analysis to provide an updated overview of knowledge representation tools relevant to exposure research. The work by [21] creates a new semantic resource for exposures, which is evaluated both in a clinical setting and on scientific literature. The resource contains (i) manual annotations derived from clinical notes and knowledge from the Unified Medical Language System (UMLS) to find exposome concepts. Ref. [20] use five corpora of epidemiological studies with different exposures and outcomes to extract exposure-related information that can aid systematic reviews and other summaries. In this work, a rule-based system called GATE [63] is used that relies on the development of dictionaries, where a total of 21 dictionaries were manually created with domain-specific exposures and outcomes. Research conducted by [19] uses rule-based patterns to analyse 60 PubMed abstracts in the obesity domain for six semantic concepts (study design, population, exposures, outcomes, covariates and effect size). Fourteen separate dictionaries are created that contain terms related to the previously mentioned six semantic concepts using a variety of tools [64,65]. Research conducted by [27] enhances the existing METLIN Exposome database to include over 950,000 unique small molecules. As part of this work, IBM Watson [66] is utilised, where Watson’s NLP approach is based on both rules (e.g., dictionary) and machine learning. The authors of [40] developed a rule-based SES (socioeconomic status) algorithm (https://github.com/vserch/SES (accessed on 12 November 2021)) to analyse Electronic Health Records using the Ruby programming language. In this work, the effects of socioeconomic factors on overall health (e.g., mortality, education, occupation) in minorities are used to ensure that these factors will be considered as exposure in future work. In summary, we found that common knowledge sources are dictionaries, lists and ontologies, where sources for this knowledge often are existing literature or clinical notes. Interestingly, there is not one preferred text mining tool used in any of the studies, and therefore, a large variety of different NLP tools are utilised.

## 3.3. Database Creation and Fusion

One of the most popular databases created is the comparative toxicogenomics database (CTD), which was developed in 2004 and is updated annually [45]. Generally speaking, this resource is made up of three databases, which include (i) a third party database that contains data from external sources (e.g., MeSH), (ii) a manually curated database of data screened by scientists and (iii) a public web application that combines data from the curation database and third party database. The resources’ aim is to provide content that relates chemical exposures with human health to gain a better insight into diseases that are influenced by the environment. Research by [33] created an updated human exposome database for predicting the biotransformation of chemicals by using literature mining to manually identify scientific articles. For this work, PubMed was queried based on several keywords related to the exposome (e.g., human exposome, drinking water, air, and disinfection or combustion by-products), where most selected publications were review articles that contain environmental matrices (e.g., indoor air exposome, dust exposome, or waterborne chemicals). The work by [34] uses the text mining approach proposed by [36] to generate a new database of organic pollutants in China. The database is based on 2799 scientific publications and includes a total of 112,878 records. Research conducted by [46] uses the AOP-helpFinder tool as proposed by [36] to screen a PubMed corpus for exposure to endocrine-disrupting chemicals. The authors of [35] utilise text mining in combination with integrative systems biology to support decision making for the usage of BPFs (bisphenol F) in manufacturing and therefore circumvent adverse outcome pathways (AOP). To establish a connection between environmental exposures (e.g., to BPFs) and health effects, a variety of existing literature and databases such as PubMed, ToxCast, CompTox, and AOP-wiki are used. In this work, a previously proposed text mining tool called AOP-help Finder [60] is used to analyse abstracts for links between chemical substances and AOPs. The corpus for this work was developed using both automatic and manual searches. First, an automatic search of PubMed was conducted using the AOP-helpFinder tool to identify links between BPF and AOPs. Then, TOXLINE [67] was searched from the year 2017 for articles that contain BPF and synonyms of BPF in a toxicological context. The authors of [47] present an update of the environmental exposure to the nanomaterials database by using NLP to retrieve information from textual data and integrate it into the database. The first step in this work is to use OpenNLP (https://opennlp.apache.org/ (accessed on 19 November 2021)) to preprocess and prepare a corpus of 10 scientific articles related to environmental risk assessment. An ontology called EXPOSEO ontology is subsequently developed and used to match the extracted information into concepts that can be integrated into the existing database. The work by [36] uses text mining to create a list of all chemicals related to ‘blood-associated chemicals’, which is then used to create a Blood Exposome Database. Several keywords were used to query PubMed, where the results were then checked manually to remove false positives and a phrase exclusion list was created. The final number of literature abstracts found is 1,085,023 (https://exposome1.fiehnlab.ucdavis.edu/download/pmid_title_abstract_sb.zip (accessed on 19 November 2021)) and then linked to chemicals, based on the synonym for a chemical, existing links between PubChem and PubMed and by mining supplement tables for chemical synonyms using R (Code in R: https://github.com/barupal/exposome (accessed on 19 November 2021)). As a result, new blood chemicals were discovered in the literature. A similar approach for assessing cancer hazards was used by [68] using the PubMed literature. The work by [69] uses a three-step process to update the comparative toxicogenomics database (CDT) with exposure data from the scientific literature sourced on PubMed. A variety of techniques are used to extract vocabularies, which include but are not limited to MeSH [70], Gene Ontology [71] and NCBI Gene [72]. These techniques extract vocabularies for chemical and anatomy words, disease terms, biological processes and geographic locations, respectively. Finally, the data are integrated into the CDT, creating 49 new tables that contain 239 columns. Research by [37] proposes a new database called the Toxin-Toxin-Target Database (T3DB), which consolidates multi-disciplinary data on toxic compound exposure. A taxonomy of compounds is generated using a classifier to categorise compounds into groups, and then, an ontology of chemical entities is developed. In a nutshell, we find that there is a need for and high usage of databases that hold domain-specific knowledge for exposure research. Furthermore, most databases outlined in this review are generated using literature mining or existing databases, where information commonly retrieved include chemicals, anatomy words, disease terms, biological processes and geographic locations.

## 3.4. Literature Reviews and Qualitative Research

Ref. [38] conducts a review of existing ontologies relevant to the external exposome research and argues for the future development of semantic standards. This argument is driven by the variation of exposome resources, where differences include but are not limited to variables having the same or similar names but measuring different exposures. The work by [26] produces a systematic literature review on transport-related injury, where the first reviewer used traditional methods and the second reviewer utilised text mining techniques to perform the same review. The text mining portion of this work uses WordStat [73], QDA Miner [74], and literature screening was conducted in Abstrackr [75]. Research by [39] investigates how the public reacted to reports of increased lead levels in school drinking waters. Both a quantitative and qualitative evaluation was performed, where it was found that (i) the majority of tweets were by news agencies and people holding positions in public offices, and (ii) the three most important themes of tweets were information sharing, health concerns and socio-demographic disparities. Overall, we have found that there is a small number of existing reviews that include the use of NLP methods and tools in exposure research. In addition to this, there is also a utilisation of mixed methods to better gauge public opinion on exposure-related health concerns.

## 4. Discussion

There are a number of challenges remaining in the field of NLP applied to occupational exposure research. In the following section, we outline some challenges and opportunities for future work in this area:

- **Data volume and quality** Whilst there has been some use of unsupervised machine learning methods (e.g., clustering via LDA) in the selected studies, a majority use supervised machine learning. One downside of this is that the latter approach requires human annotated data, which usually requires expert knowledge and is therefore a time-consuming and costly process. To overcome this issue, the use of semi-supervised or unsupervised learning methods might be explored, because it requires either significantly less annotated training data or none at all. An example of this is the use of topic modelling techniques to cluster jobs and exposures from the existing literature. Another opportunity lies in using semi-supervised Named Entity Recognition to increase the coverage of annotated literature.
- **Novel deep learning techniques** The present studies predominantly utilise traditional machine learning techniques (e.g., SVMs); however, the field has drastically evolved over recent years with more advanced techniques known as deep learning methods producing scaleable and accurate results. This includes but is not limited to transfer learning [76] or adversarial learning [77], which include a variety of neural networks structures or knowledge graphs that have been at the core of NLP research.
  This also includes Transformer-based methods [78] (e.g., large pre-trained language models such as BERT [79]), which have made a significant impact on the field of NLP over recent years and could prove to be useful in NLP for occupational exposure research. This type of deep learning method is based on attention [80], which has been shown to improve results in a variety of other domains that have utilised NLP (e.g., healthcare). These advances could be used to improve tasks such as Named Entity Recognition (NER) [81] or Relation Extraction (RE) [82] in occupational exposure research, which up until this point have relied on traditional machine learning only. Both tasks could prove useful in the context of occupational exposure research to automatically identify key concepts (e.g., types of exposures, jobs or work environments) but also how they relate to one another (e.g., a particular role is in a specific work place). Other advances could be made through the use of unsupervised methods, which thus far have also relied on traditional machine learning only. More recent methods such as Neural Topic Models (NTM) have become increasingly popular for different tasks, including document summarisation and text generation [83] due to their flexibility and capability. These methods could also be applied to occupational exposure research to uncover new topics and concepts at a larger scale or draw new connections between exposures and work environments. Similarly, NTM methods could also be coupled with pre-trained language models to further boost performance and result in more accurate representations of new topics [83].
- **Extrapolating existing research to other domains of exposure research** Most of the research explored in this review is specific to a particular type of exposure, databases or enhancement of literature reviews. The domain-specificity and different needs/requirements for each type of exposure make it therefore hard to extrapolate these existing works to other fields, link and scale up existing approaches.

## 5. Conclusions

In this work, we have manually reviewed 37 papers relevant to NLP applied to occupational exposure research. Our results show that (i) there has been an increase in articles published, (ii) most work uses existing NLP tools, and (iii) traditional machine learning is the most popular approach. Furthermore, we have outlined challenges and opportunities for future research that could further advance the field.

## Figures and Tables

**Table 1:** A categorisation of each paper based on *tools used*, *resources* and *computational methods*.

	Papers
**Tool used**	
NLTK	[9,10,11,12,13],
	[14]
Other	[9,15,16,17,18],
	[19,20,21],
	[13,22,23,24],
	[25,26,27]
Not declared	[15,28,29,30,31,32],
	[33,34,35,36,37],
	[38,39,40]
**Resources**	
Scientific literature	[12,15,28,29,41,42],
	[14,22,23,31],
	[24,43,44],
	[19,20,21,33,34,45],
	[35,36,46,47]
Existing Database	[13,30,35,37,45]
Twitter	[11,18,39]
EHR	[9,21,48]
Accident reports	[10,17,25]
**Computational Method**	
Machine learning	[9,15,28,41],
	[10,12,28,29],
	[13,17,30,42,48],
	[11,14,18,22,31],
	[23,24,25,27]
Knowledge based	[19,20,21,43,44]
Database creation and fusion	[27,33,34,35,45,46],
	[36,37,47]
Rule-based algorithms	[27,40]

## Data Availability

Not applicable.

## Abbreviations

The following abbreviations are used in this manuscript:

AI	Artificial Intelligence
AOP	Adverse Outcome Pathways
BERT	Bidirectional Encoder Representations from Transformers
CTD	Comparative Toxicogenomics Database
DRS	Document relevancy score
LDA	Latent Dirichlet Allocation
LSA	Latent semantic analysis
LSTM	Long Short Term Memory
NER	Named Entity Recognition
NLP	Natural Language Processing
NLTK	Natural Language Toolkit
NTM	Neural topic models
PCA	Principal component analysis
RE	Relation Extraction
SVM	Support Vector Machine
TF-IDF	frequency–inverse document frequency
UMLS	Unified Medical Language System
